# High-voltage aqueous zinc-ion batteries with conversion-type anode and modified electrolyte

**DOI:** 10.1093/nsr/nwae229

**Published:** 2024-07-04

**Authors:** Junnan Hao, Shi-zhang Qiao

**Affiliations:** School of Chemical Engineering, The University of Adelaide, Australia; School of Chemical Engineering, The University of Adelaide, Australia

Aqueous zinc-ion batteries (AZIBs) with near-neutral electrolytes have attracted wide attention for their high safety, low cost and environmental friendliness, making them a promising candidate for sustainable energy storage. However, the development of high-voltage AZIBs has been hindered by the limited choice of anode materials and the interfacial incompatibility between the electrode and the electrolyte [1]. In addressing these challenges, Fang and his team have made a breakthrough by introducing conversion-type anode chemistry tailored for high-voltage near-neutral AZIBs [2].

**Figure 1.**
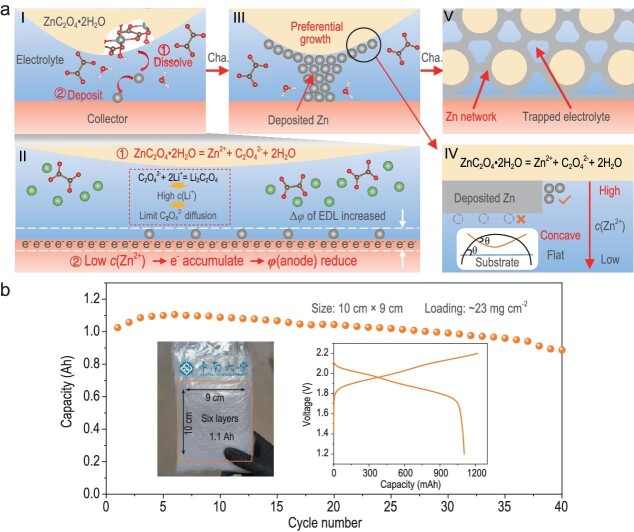
(a) Stages of the Zn and ZnC_2_O_4_·2H_2_O conversion reaction: initial (inset I), growth (inset III) and final (inset V). Initial stage includes dissolution and deposition (inset II). Inset IV explains the preferential growth in inset III, focusing on the Zn²^+^ concentration and substrate differences. (b) Cycling stability of a six-layer pouch cell (10 cm × 9 cm) at 0.44 mA cm^–^^2^. The inset shows the 1.1-Ah-capacity pouch cell device and its charge/discharge curve. Adapted with permission from Ref. [2].

This conversion-type anode is based on a reversible reaction between ZnC_2_O_4_·2H_2_O particles and 3D Zn metal networks (Fig. 1a). Unlike the traditional SO_4_^2^^–^-based electrolyte, the CH_3_COO-based electrolyte demonstrates a lower charge voltage and higher discharge voltage when coupled with a LiFePO_4_ cathode. Molecular dynamics simulations confirm that the CH_3_COO-based electrolyte has a lower percentage of hydrogen bonding compared with the SO_4_^2^^–^-based electrolyte, indicating reduced water activity and suppressed hydrogen evolution. It is noticed that this conversion-type anode with a CH_3_COO-based electrolyte ensures compatibility with diverse battery systems, including those using sodium and iodine ions.

Furthermore, the study introduces a crowded micellar electrolyte with a water confinement effect, which is crucial for maintaining the stability and reversibility of the cathode at operating voltages of >2.0 V. The crowded micellar electrolyte significantly suppresses electrolyte decomposition and cathode particle cracking, enabling a high-capacity retention of 95% after 100 cycles. This approach addresses the intrinsic narrow electrochemical stability of aqueous electrolytes, which has traditionally limited the operating voltage of AZIBs.

In addition to stabilizing the electrolyte/electrode interface, Fang *et al.* explored the technological challenges from coin cell configurations to Ah-scale pouch cells. These challenges include the sluggish kinetics of solid–solid electrode reactions, capacity excitation under high loading of active materials and complexities in preparing large-area quasi-solid electrolytes. Remarkably, the study achieved an 88% capacity retention under a high loading of >20 mg cm^–^^2^ in practical pouch cells, demonstrating the feasibility of scaling up this technology for real-world applications (Fig. 1b). The successful fabrication of a 1.1-Ah-level pouch cell highlights the practical potential of this innovative battery design.

In summary, this work bridges the gap between theoretical research and practical application, offering a scalable solution for sustainable energy storage and emphasizing the importance of innovative conversion-type electrode design and electrolyte engineering.
